# Esophageal Motility Disorders in the Natural History of Acid-Dependent Causes of Dysphagia and Their Influence on Patients’ Quality of Life—A Prospective Cohort Study

**DOI:** 10.3390/ijerph182111138

**Published:** 2021-10-23

**Authors:** Joanna Sarbinowska, Benita Wiatrak, Dorota Waśko-Czopnik

**Affiliations:** 1 Department of Gastroenterology and Hepatology, Wroclaw Medical University, Borowska 213, 50-556 Wroclaw, Poland; dorota.wasko-czopnik@umed.wroc.pl; 2 Department of Pharmacology, Wroclaw Medical University, Mikulicza-Radeckiego 2, 50-345 Wroclaw, Poland; benita.wiatrak@umed.wroc.pl

**Keywords:** diagnostic delay, esophageal motility disorders, gastrointestinal quality of life index, high-resolution manometry

## Abstract

Background: Esophageal dysmotility may be the cause or a secondary effect of gastric acid-dependent diseases: erosive reflux disease (ERD), Schatzki ring (SR) and eosinophilic esophagitis (EoE). Methods: This study aims to compare concomitant dysphagia with ERD, SR and EoE, considering manometric patterns, their role in the natural history and their impact on assessing quality of life. Fifty-eight patients with dysphagia underwent high-resolution manometry and esophago-gastro-duodenoscopy (EGD) with an assessment of SR, ERD and sampling for EoE, completed a questionnaire with the Eating Assessment Tool (EAT-10) and the Gastrointestinal Quality of Life Index. Based on endoscopic images and the histopathological criterion of EoE (≥15 eosinophils/high-power field), patients were assigned to groups with ERD, EoE, SR and with normal endoscopic and histopathological images. In the data analysis, *p* ≤ 0.05 was considered statistically significant. This trial was registered with ClinicalTrials.gov (no. NCT04803162). Results: Both EoE, SR and ERD correlate with ineffective motility. In ERD, normal peristalsis precedes the development of the disease, unlike EoE, which develops later and leads to absent contractility. The development of SR is associated with disorders of the upper esophageal sphincter (UES). In the group with SR and ERD, UES insufficiency significantly reduces the quality of life. Patients with normal esophagus in EGD scored the lowest quality of life and those with SR had the most severe dysphagia. Conclusion: The esophageal motility disorders co-occurring with endoscopic and histological anomalies do not significantly affect the severity of dysphagia, however, in the case of patients with ERD and SR and concomitant UES insufficiency, this motor dysfunction has a significant impact on the reduction in the patients’ quality of life. Although no specific esophageal motility pattern typical of EoE, ERD and SR has been identified, comparative assessment of manometric features may have a potential role in differential diagnosis.

## 1. Introduction

Dysphagia, defined as a swallowing disorder consisting of difficulty in biting food and moving it towards the throat through the esophagus into the stomach, affects every 17th person in the world during their lifetime [1]. Complications related to dysphagia increase healthcare costs, and its chronic and recurrent nature, necessitating the need for invasive diagnostic tests, significantly reduces the quality of life of patients [2].

Both in Western Europe and North America, with the spread of proton pump inhibitors (PPIs), the incidence of GERD’s complications as a cause of esophageal dysphagia is decreasing in favor of eosinophilic esophagitis (EoE), which is increasingly recognized as the main cause in children, as well as in adults [1].

Until recently, responses to food and inhalant allergens have been the main contributors to the pathophysiology of chronic EoE [3]. However, it is currently known that damage to the esophagus caused by hydrochloric acid, and the resulting discontinuance of the integrity of the epithelium, may favor the penetration of the allergen [4,5]. Thus, the relationship between GERD and EoE can be bidirectional and complex. Moreover, the coexistence of these two disease entities may constitute a specific perpetual motion machine in the activation of the chronic inflammatory response and the intensification of clinical symptoms.

Although dysphagia is sometimes associated with uncomplicated GERD, its presence raises the suspicion of endoscopic macroscopic changes in the form of erosive esophageal reflux disease (ERD) or stenosis, neoplastic infiltration or rings [6,7]. The pathophysiology of the formation of the lower esophageal ring, also known as the Schatzki ring (SR), is usually associated with chronic GERD and is intended to be a natural form of self-protective response against exposure to the acid reflux, thus minimizing symptoms and preventing the development of other complications such as Barrett’s esophagus [8,9,10]. According to the available literature data, GERD justifies the development of SR in only two-thirds of patients [11]. The etiology in the remaining patients is unknown or hypothetically related to the advanced process of esophageal fibrosis and trachealization in the course of EoE [10].

In the etiology of dysphagia, which is a symptom of these diseases, esophageal motility disorders are also important, as it may be the cause and predictor or be a secondary effect and complication of acid-dependent esophageal diseases. However, the manometric findings in patients with EoE, ERD and SR are inconsistent and ambiguous [12,13,14]. Thus far, the significance of the possible coexistence of manometric disorders with the discussed esophageal diseases in the context of decreased quality of life of patients with these disorders has not been assessed.

Therefore, this study focuses on the comparative characteristics of dysphagia in a population of patients diagnosed with gastric acid-related esophageal diseases: ERD, EoE and SR, considering manometric patterns and the quality of life of patients assessed by the Gastrointestinal Quality of Life Index (GIQLI) questionnaire [15]. The study aimed to evaluate the significance of the coexistence of manometric disorders and acidic esophageal diseases and their role in shaping the assessment of patients’ quality of life.

## 2. Materials and Methods

### 2.1. Study Population

Patients referred for the diagnosis of dysphagia to the Department of Gastroenterology and Hepatology and the Department of Otolaryngology, Head and Neck Surgery at the Medical University of Wroclaw were recruited to participate in a two-center prospective cohort study. From 1 November 2017 to 4 April 2020, 58 patients were enrolled in the project. The exclusion criterion from the survey was already diagnosed chronic diseases with possible eosinophilic infiltration of the gastrointestinal tract, rheumatological, dermatological and genetic disorders with possible peripheral eosinophilia and neoplastic infiltration of the esophagus. All project participants gave their written consent to participate in the study, and the ethical consent to implement the project was obtained by the Bioethics Committee of the Medical University of Wrocław on 17 August 2017 (KB No. 544/2017), with another extension on 6 December 2018 (KB No. 730/2018).

### 2.2. Endoscopy and Specimen Collection

All project patients underwent esophagogastroduodenoscopy (EGD) with an assessment of the presence of SR, defined as a peripheral thin membranous ring of the mucosa located above the gastroesophageal junction [16]. During the examination, the presence of endoscopic esophagitis was also monitored (the severity of ERD was assessed according to the Los Angeles classification) [7]. Regardless of the presence of macroscopic changes in the esophagus, six additional pieces of biopsies (two distal, medial and proximal) were taken to assess the maximum number of eosinophils per high-power field (HPF). Two independent pathologists assessed all samples, while EGD was performed by one gastroenterologist using Olympus devices (GIF-Q180).

Based on endoscopic images and the histopathological criteria for the diagnosis of EoE, that is, the presence of ≥15 eosinophils/HPF, patients were assigned to the following groups: group 1, patients with ERD; group 2, patients with EoE; group 3, subjects with SR. The patients excluded from the above diagnoses constituted a group of patients with dysphagia without endoscopic and histopathological abnormalities.

### 2.3. High-Resolution Manometry

In order to avoid the therapeutic effect of the endoscope, and possible influence on manometric parameters and esophageal motility assessment in all patients, HRM was performed before esophagogastroduodenoscopy. HRM was performed, in patients who fasted for 8 h, in the supine position and after prior nasal anesthesia with lignocaine gel. After pre-calibration, a catheter with 36-solid state circumferential sensors spaced at 1 cm intervals (Sierra Scientific Instruments, Los Angeles, CA, USA) was inserted through the patient’s nose into the stomach. After 3–5 min of rest, basal sphincter pressure (landmark ID frame) was assessed for another 30 s, followed by 10 sips of 10 mL water (swallow frames) and in the end, a Multi Rapid Swallow Test (MRS-test). High-resolution manometry data were recorded and analyzed using the ManoViewTM ESO 3.0 software (Sierra Scientific Instruments). Interpretation of the results was made according to the Chicago classification 3.0 [17].

### 2.4. Characteristics of Dysphagia and Gastrointestinal Quality of Life Index

In addition to the assessment of esophageal motility and the endoscopic and histopathological findings, the participants included in the project also completed a questionnaire on age, sex, marital status, place of residence, weight and height, smoking and alcohol consumption, possible chronic diseases—including atopic diseases—and diagnostic delay of dysphagia (interpreted as the time elapsed between the first episodes of dysphagia and the clinical diagnosis). In order to estimate the severity of dysphagia, the Eating Assessment Tool (EAT-10) questionnaire was used [18], consisting of 10 statements concerning problems with swallowing and subject to a 5-step assessment by the surveyed patients (where 0 points means no problem and 4 points—hard problem). Each of the individual statements and the total value made it possible to compare the severity of dysphagia in the studied groups of patients. To expand the EAT-10 questionnaire, patients in this study also reported the presence and severity of heartburn, regurgitation and globus according to the above criteria. However, the score obtained for the presence of these symptoms was considered separately and was not summed up with the total EAT-10. The standardized GIQLI questionnaire was used to assess the patients’ quality of life, containing 36 questions about the symptoms of gastrointestinal diseases and related symptoms [15]. In terms of GIQLI, 5 measurement domains were distinguished: concerning symptoms (19 questions: No. 1–9 and 27–36), emotional state (5 questions: No. 10–14), physical functions (7 questions: No. 15–21), social functions (4 questions: No. 22–23 and 25–26) and treatment outcomes (1 question: No. 24). As the present study did not intend to intervene, the area of patient’s treatment was not analyzed separately. For each of the 36 questions contained in the GIQLI, the respondent answered from “all of the time” to “never,” with 0 to 4 points, respectively. Then, the results of individual questions were summarized, and the obtained result could reach a maximum of 144 points, which was interpreted as the best quality of life. The results for individual GIQLI domains were calculated as the arithmetic mean of points obtained in the questions included in domains [15].

### 2.5. Statistical Analysis

In statistical analysis, chi^2^ tests were used to evaluate qualitative data. Quantitative data were used for Kruskal–Wallis ANOVA calculations with appropriate post-hoc. The correlation was calculated using Spearman ranks. The point of significance was *p* ≤ 0.05.

## 3. Results

### 3.1. Characteristics of the Population

By applying the exclusion criteria, 58 patients with dysphagia were eventually enrolled in the study. Based on endoscopic examinations and histopathological evaluation of specimens taken from the esophageal mucosa, in as many as 58.62% of cases the cause of dysphagia reported by patients was not found. ERD was diagnosed in 7, SR in 8 and EoE in 16 patients. The disproportions in the size of the groups did not significantly affect the differences in demographic characteristics and disease burden, including atopic burden (Table 1). Among comorbid non-atopic diseases reported by project participants, the most common were hypothyroidism (10.34% of patients), hypertension (5.17%), insulin resistance (3.45%), polycystic ovary syndrome (3.45%) and tetany (3.45%), as well as in single patients: Wilson’s disease, microscopic colitis, psoriasis, osteoarthritis, chronic sinusitis, paroxysmal tachycardia and irritable bowel syndrome. The lack of coexistence of many gastroenterological disorders, apart from the assessed swallowing disorders, made it possible to use the GIQLI questionnaire as a reliable tool for assessing and comparing the quality of life of the studied population. 

Despite the demographic homogeneity of the project participants, statistically significant (*p* < 0.001) differences were observed in the manometric diagnoses included in the Chicago classification and in the assessment of the function of the upper esophageal sphincter (UES) and esophagogastric junction (EGJ). There were no cases of achalasia, distal esophageal spasm or jackhammer esophagus in the studied group of patients. In patients diagnosed with SR, absent contractility was diagnosed more often than in the other groups, and in the case of EoE, lower esophageal sphincter (LES) insufficiency. Patients with ERD in the study population more often than in the other groups of patients had ineffective motility (IEM), UES insufficiency and manometric features of hiatal hernia, while in patients with normal findings in endoscopic and histopathological examinations, EGJ outflow obstruction, UES spastic disorders and normal peristalsis were more often found (Table 1).

Statistically, differences between the patient groups were also found regarding the characteristics and severity of dysphagia assessed using the EAT-10 questionnaire (Table 1). Patients diagnosed with SR significantly more often reported difficulty in swallowing fluids (*p* = 0.03), odynophagia (*p* = 0.04) and rated the severity of dysphagia in summary EAT-10 assessment as the highest compared to other groups of patients (*p* = 0, 05). Swallowing stress was most commonly reported in patients without macroscopic and microscopic esophageal abnormalities (*p* = 0.001) and in patients diagnosed with ERD (*p* = 0.04). Heartburn was characteristic of the ERD group, while regurgitation and globus were more common in SR patients.

The subjective assessment of the quality of life of patients in terms of symptoms, physical and emotional well-being resulted as significantly statistically different between the groups of patients and it was assessed the worst in the group of patients with EoE (Table 1).

### 3.2. Manometric Patterns of Eosinophilic Esophagitis, Erosive Esophagitis, Schatzki Ring

Despite the evaluation of possible correlation between endoscopic, histopathological and manometric diagnoses, no unequivocal motor patterns differentiating esophageal acid-dependent disorders have been established. The moderate positive correlations with IEM were observed for EoE, SR and ERD, although statistical significance was obtained only for ERD (*p* = 0.04). Additionally, the weak, negative, statistically significant correlation of IEM with the normal endoscopic and histopathological image of the esophagus allows classifying this manometric diagnosis as a predictive factor in differentiating the coexistence of other macro- and microscopic disorders (Table 2).

Among the diseases of the esophageal sphincters and the EGJ, the presence of a hiatal hernia weakly, positively, but statistically significantly correlated with ERD diagnosis and weakly positively, though slightly, with SR. Based on the obtained, although statistically insignificant, correlation systems of UES pressure disorders, it can be assumed that spasticity is associated with a normal endoscopic image and insufficiency with other accompanying diseases of the esophagus (Table 2).

### 3.3. Diagnostic Delay and the Occurrence of Manometric Disorders—“What Was Earlier—An Egg or a Hen?”

Among study participants, 51.72% of people stated that the diagnostic delay, defined as the time elapsed from the onset of the first episodes of dysphagia to the time of first diagnosis, was over six months, of which 43.75% were in the group of patients with EoE, 71.43% of patients with ERD and 87.50% of patients with SR (Table 3).

The length of the diagnostic delay of dysphagia correlates positively with the diagnosis of SR and ERD. Still, in the case of SR, this correlation is stronger and statistically significant (*p* = 0.03)—Table 4.

Based on the direction of Spearman’s correlation coefficient, the potential significance of esophageal manometric disturbances in the natural course of gastro-related diseases was assessed (Table 5). In ERD, normal esophageal peristalsis seems to significantly precede the development of the disease (*p* = 0.05), while the consequence is significantly more often IEM (*p* = 0.001). LES and UES insufficiency and hiatal hernia are potential predictors of the development of both ERD and EoE, with the two disorders appearing to differ in the motility of the esophageal body. Already existing motility disorders much more often precede the development of EoE: IEM (*p* = 0.02) and EGJ outflow obstruction (*p* = 0.04), and the absent contractility (*p* = 0.02) develops with time as the disease progresses. In the development of SR, esophageal sphincters dysfunction appears to be significant—LES insufficiency (*p* = 0.04) and UES spastic disorders (*p* = 0.04), while UES insufficiency is secondary (*p* = 0.03). In the group of patients without diagnosis on endoscopic and histopathological examinations, no significant strong relationships between diagnostic delay and manometric disorders were observed (Table 5).

### 3.4. The Coexistence of Manometric Disorders Exacerbates Dysphagia and Reduces the Quality of Life in Patients with Erosive Esophagitis, EoE and SR

In the Spearman’s rank correlation analysis, no statistically significant associations were found between the dysphagia assessment measured with EAT-10 and the coexistence of esophageal motility disorders.

Moreover, there was no significant reduction in quality of life measured as total GIQLI due to the coexistence of esophageal body motility disorders in any of the patient groups studied (Table 6).

Statistically important values were obtained only regarding the coexistence of UES insufficiency with SR (*p* = 0.01) and ERD (*p* = 0.01). To deepen the analysis of the impact of UES insufficiency on the worsening of quality of life in these patient groups, the coexistence of esophageal body manometric abnormalities assessed by Chicago classification correlated with this disturbance in the proximal part of the esophagus. There was a statistically significant correlation between UES insufficiency, abnormal motility (*p* = 0.01) and IEM (*p* = 0.03) in the group of SR patients. However, this observation was not confirmed in ERD patients without the presence of SR (Table 7).

## 4. Discussion

Although the first published description of the lower esophageal ring by Richard Schatzki dates back to 1953 [19], in the early 1990s EoE was defined as a distinct clinicopathological syndrome [4], and about 15 years ago the current Montreal definition and classification of GERD was established [20], acid-related esophageal diseases continue to pose both a scientific and clinical challenge. This work, through a comparative assessment of esophageal motility disorders accompanying acid-dependent causes of dysphagia and their location in the natural history of these diseases, allows for a broader understanding of pathophysiology and improvement of management by indicating a new path of differential diagnosis.

Thus far, no specific motility patterns of acid-dependent diseases have been identified, but HRM invariably remains an integral part of the diagnosis of dysphagia [21]. In the case of GERD with symptoms resistant to treatment, it is performed to qualify for anti-reflux surgery—so it can not only explain the cause of symptoms by assessing the function of the EGJ, the presence of a hiatal hernia or LES incontinence but it may condition making therapeutic decisions [13]. It is estimated that in approximately 5.7% of cases, underdiagnosed SR or EoE may be responsible for the presence of EGJ outflow obstruction. Still, the exact influence of SR on the HRM picture has not been studied thus far [14]. The main topic of interest of manometrists in recent years is assessing the importance of HRM in the diagnosis and monitoring of EoE, a less invasive method if compared to EGD with the collection of specimens for histopathological evaluation [12]. In EoE, dissociation occurs in the contraction of the longitudinal and circular muscles during primary peristalsis, most likely in response to eosinophilic infiltration and tissue remodeling leading to fibrosis [22]. Thus far, eight studies have been published regarding a possible correlation between different GERDs manifestations and specific manometric patterns. In short, they have demonstrated that: the manometric pattern of patients with PPI-responsive esophageal eosinophilia was similar to the motility of patients with GERD [23]; inflammatory and fibrostenotic EoE phenotypes were differentiated based on intrabolus pressure (values significantly higher in the case of the fibrostenotic phenotype) [24]; the resolution of manometric disturbances was confirmed after topical treatment with budesonide [25]; there is a correlation between increased distal contractile integral (DCI) and the severity of symptoms [26]. Moreover, EoE was associated with increased pan-esophageal pressurization [27], weak and failed peristaltic integrity worsening with the duration of the disease [28], as well as with achalasia and obstructive motor disorders [29]. Two studies did not identify an EoE-specific esophageal motility disorder correlating with the endoscopic picture and the severity of dysphagia [25,30]. The obtained ambiguous and inconsistent test results have not allowed HRM in diagnostic guidelines for any of the causes of acid-related esophageal dysphagia.

Similarly, this project failed to define esophageal motility patterns that would be unambiguous for the diseases studied. On the other hand, a significant relationship has been demonstrated between ERD and IEM development secondary to the advancement of erosive lesions in the duration of the disease. The opposite situation to ERD arising in the esophagus with normal motility of the body was observed in EoE, where IEM and EGJ outflow obstruction frequently preceded the development of the disease, while the disorders correlated with the duration of esophageal diseases consisted of absent contractility. It can be assumed that the presence of manometric disturbances preceding the result of the inflammatory reaction somehow programs the further path of its development. In the case of ERD, it depends on the action of acid. However in EoE, it also depends on food allergens, which due to motility disorders have prolonged contact with the esophageal mucosa [31]. Hiatal hernia was significantly associated with ERD, which was not found in SR. The development of SR was significantly preceded by LES insufficiency. This is justified by the very pathophysiology of the ring, which is a kind of mechanical protective barrier against exposure to further esophageal reflux of irritating gastric contents, exacerbated by LES insufficiency [8]. However, a previously unknown observation is the coexistence of UES disorders in the natural history of SR—the primary role of spastic disorders and secondary UES insufficiency. Hypothetically, spastic disorders may constitute an attempt at self-protective synergy with SR, against the action of irritating gastric contents, while UES insufficiency secondary to the development of the SR and arising with the duration of the disease may result from a decrease in resting pressure due to sphincter fatigue or from a reduction in the role of the sphincter secondary to a significant narrowing of the esophageal lumen by the ring.

When assessing the impact of manometric disturbances on the quality of life of patients with endoscopic causes of dysphagia, it was the UES insufficiency that had a significant bearing on the subjective assessment of GIQLI in patients with ERD and SR. The hypothesis that the effectiveness of UES affects the efficacy of peristaltic wave propagation, and thus the act of swallowing itself, which may affect the assessment of the quality of life, was confirmed only in the group of SR patients. In this group the correlation of UES disorder with the presence of other manometric abnormalities of the esophageal body was confirmed, including the development of IEM.

Undoubtedly, more than half of the project participants who did not have the cause of dysphagia identified in the endoscopic and histopathological examination, and especially the patients without manometric abnormalities (over a quarter of this group of respondents), definitely require a more in-depth analysis. Patients with no justification for dysphagia on endoscopic and histopathological examinations reported stress associated with swallowing in the EAT-10 questionnaire more often than in other patients. They also assessed the quality of life the lowest in the GIQLI questionnaire. Moreover, in the comparative assessment, although without statistical significance, UES spastic disorders were found in this group of patients on the manometric test. Such disorders, despite the lack of unambiguous literature data, are associated with the globus symptom and increased mental tension [32,33]. The lack of justification for dysphagia in this group of patients may also result from a limitation of the project, such as the lack of pH-metry with impedance in the diagnosis of symptoms reported by patients, which would probably allow us to objectively select a group of patients with non-erosive esophageal reflux disease (NERD) [6].

## 5. Conclusions

In the light of the literature reports to date and the results of this project, it seems impossible to identify specific esophageal motility patterns typical for EoE, ERD and SR. In this project, the esophageal motility disorders co-occurring with endoscopic and histological diagnoses have been shown not to significantly affect the severity of dysphagia. However, in the case of patients with ERD or SR and concomitant UES insufficiency, this motor dysfunction had a significant impact on the worsening of the patients’ quality of life.

Specific comparative assessment of manometric patterns and features, considering the impact of diagnostic delay and participation in shaping the quality of life of patients, can help to improve the differentiation of coexisting and often overlapping causes of acid-dependent dysphagia.

## Figures and Tables

**Table 1:** Demographic and clinical characteristics of participants.

Parameters		All Patients	ERD	SR	EoE	Normal Esophagusin EGD and Histopathology	*p*
Patients [n (%)]		58 (100%)	7 (12.1%)	8 (13.8%)	16 (27.6%)	34 (58.6%)	*p* = 0.00001
Age median (range)		34 (20–68)	43 (20–60)	49 (24–68)	28.5 (20–50)	33.5 (24–63)	*NS*
Demography male gender [n (%)]		30 (51.7%)	0	2 (25.0%)	5 (31.3%)	24 (70.6%)	*NS*
Marital single status [n (%)]		11 (19.0%)	2 (28.6%)	1 (12.5%)	4 (25.0%)	8 (23.5%)	*NS*
Place of residence—a city with over 100,000 inhabitants [n (%)]		35 (60.3%)	4 (57.1%)	4 (50.0%)	9 (56.3%)	20 (58.8%)	*NS*
BMI median (range)		23.09 (16.26–33.3)	24.25 (22.39–26.57)	24.58 (21.09–29.41)	24.09 (19.13–28.08)	22.44 (16.26–33.30)	*NS*
Smoker [n (%)]		9 (15.52%)	2 (28.6%)	0	6 (37.5%)	4 (11.8%)	*NS*
Alcohol consumption [n (%)]		35 (60.3%)	6 (85.7%)	5 (62.5%)	11(68.8%)	21 (61.8%)	*NS*
Number of patients with additional non-atopic chronic diseases [n (%)]		17 (29.3%)	3 (42.9%)	1 (12.5%)	4 (25.0%)	9 (26.5%)	*NS*
Atopy [n (%)]		28 (48.3%)	2 (28.6%)	2 (25.0%)	8 (50.0%)	18 (52.9%)	*NS*
Atopy [n (%)]	inhalation allergies	14 (24.1%)	1 (14.3%)	2 (25.0%)	4 (25.0%)	9 (26.5%)	*NS*
	food allergies	9 (15.5%)	2 (28.6%)	0	3 (18.8%)	6 (17.6%)	*NS*
	bronchial asthma	5 (8.6%)	2 (28.6%)	1 (12.5%)	1 (6.3%)	2 (5.9%)	*NS*
	atopic dermatitis	5 (8.6%)	1 (14.3%)	1 (12.5%)	0	3 (8.8%)	*NS*
	allergic sinusitis	4 (6.9%)	0	1 (12.5%)	0	3 (8.8%)	*NS*
EAT-10 [mean ± SD]	1. My swallowing problem has caused me to lose weight.	0.33 (0.76)	0.43 (0.53)	0.25 (0.46)	0.25 (0.58)	0.38 (0.89)	*NS*
	2. My swallowing problem interferes with my ability to go out for meals.	1.19 (1.23)	0.71 (0.76)	1.50 (1.20)	1.56 (1.21)	1.18 (1.29)	*NS*
	3. Swallowing liquids takes extra effort.	0.60 (1.04)	0.71 (0.95)	1.12 (1.25) *p* = 0.03	0.44 (0.89)	0.62 (1.13)	*NS*
	4. Swallowing solids takes extra effort.	1.05 (1.25)	0.71 (0.95)	1.38 (1.41)	1.25 (1.34)	1.12 (1.30)	*NS*
	5. Swallowing pills takes extra effort.	1.21 (1.31)	0.86 (0.90)	1.63 (1.51)	1.50 (1.41)	1.32 (1.39)	*NS*
	6. Swallowing is painful.	0.79 (1.20)	0.86 (1.07)	1.00 (0.76) *p* = 0.04	0.56 (0.89)	0.65 (1.15)	*NS*
	7. The pleasure of eating is affected by my swallowing.	1.26 (1.04)	0.57 (0.79)	1.12 (1.36)	1.12 (1.09)	1.44 (1.05)	*NS*
	8. When I swallow, food sticks in my throat.	1.02 (1.08)	0.86 (0.90)	1.75 (0.89)	1.13 (1.09)	0.97 (1.14)	*NS*
	9. I cough when I eat.	0.97 (1.15)	0.86 (0.90)	1.25 (1.39)	1.06 (1.12)	0.91 (1.22)	*NS*
	10. Swallowing is stressful.	1.55 (0.82)	1.14 (1.07) *p* = 0.04	1.00 (0.53)	1.25 (0.86)	1.59 (0.57) *p* = 0.001	*NS*
	Total	9.97 (7.24)	7.71 (4.35)	12.00 (8.33) *p* = 0.05	10.12 (7.59)	10.18 (7.64)	*NS*
Additional symptoms [mean ± SD]	Heartburn	1.10 (1.22)	1.71 (1.50)	1.12 (1.55)	1.06 (1.34)	1.00 (1.15)	*p* < 0.0001
	Regurgitation	1.00 (1.04)	1.14 (1.35)	1.50 (1.20)	1.00 (0.97)	0.88 (1.01)	*p* < 0.0001
	Globus	1.50 (1.39)	1.43 (1.40)	2.12 (1.25)	1.38 (1.41)	1.41 (1.44)	*p* < 0.0001
Motility pattern defined per Chicago classification [n (%)]	Achalasia	0	0	0	0	0	-
	EGJ outflow obstruction	4 (6.9%)	0	0	1 (6.3%)	3 (8.8%)	*p* < 0.001
	DES	0	0	0	0	0	-
	Jackhammer esophagus	0	0	0	0	0	-
	Absent contractility	3 (5.2%)	0	1 (12.5%)	1 (6.3%)	2 (5.9%)	*p* < 0.0001
	IEM	28 (48.3%)	6 (85.7%)	6 (75.0%)	10 (62.5%)	12 (35.3%)	*p* < 0.0001
	Normal peristalsis	24 (41.4%)	1 (14.3%)	1 (12.5%)	5 (31.3%)	17 (50.0%)	*p* < 0.0001
Manometric features [n (%)]	LES insufficiency	15 (25.9%)	2 (28.6%)	1 (12.5%)	6 (37.5%)	8 (23.5%)	*p* < 0.0001
	UES insufficiency	9 (15.5%)	2 (28.6%)	2 (25.0%)	4 (25.0%)	3 (8.8%)	*p* < 0.0001
	UES spastic disorders	20 (34.5%)	2 (28.6%)	1 (12.5%)	3 (18.8%)	13 (38.2%)	*p* < 0.0001
	hiatal hernia	4 (6.9%)	2 (28.6%)	1 (12.5%)	1 (6.3%)	1 (2.9%)	*p* < 0.0001
GIQLI [mean ± SD]	Physical well-being mean points	17.00 (5.35)	18.14 (5.98)	19.50 (5.83)	19.44 (4.88)	16.47 (5.17)	*p* < 0.001
	Gastrointestinal symptoms mean points	53.72 (11.07)	56.14 (17.28)	58.50 (12.88)	58.69 (10.45)	52.88 (9.62)	*p* < 0.0001
	Social well-being mean points	12.76 (3.15)	13.71 (2.75)	13.75 (2.87)	13.81 (2.77)	12.29 (3.49)	*NS*
	Emotional well-being median points	12.10 (4.46)	15.29 (5.09)	14.37 (3.74)	14.50 (4.23)	11.18 (4.27)	*p* < 0.0001
	Total mean points	98.44 (21.02)	106.71 (30.73)	109.37 (23.63)	109.75 (18.44)	95.41 (19.30)	*p* < 0.0001

**Table 2:** Spearman’s rank correlation coefficients between diseases diagnosed based on endoscopic and histopathological examination and manometric patterns and features. Statistically significant—in red.

Esophageal Motility Disorders		ERD	SR	EoE	Normal Esophagus in EGD and Histopathology
Motility pattern defined per Chicago classification	EGJ outflow obstruction	−0.100832	−0.108866	−0.015749	0.090513
	Absent contractility	−0.086525	0.132345	0.030031	0.038154
	IEM	0.277568	0.213920	0.175691	−0.309211
	Normal peristalsis	−0.203804	−0.234547	−0.126940	0.208333
Manometric features	LES insufficiency	0.022923	−0.122062	0.164044	−0.063406
	UES insufficiency	0.133574	0.104762	0.161651	−0.220044
	UES spastic disorders	−0.046076	−0.184996	−0.204297	0.093968
	Hiatal hernia	0.316900	0.088454	−0.015749	−0.185790

**Table 3:** Diagnostic delay of dysphagia patients with ERD, SR, EoE, normal esophagus in EGD and histopathology examination.

Diagnostic Delay of Dysphagia	All Patients (n = 58)	ERD (n = 7)	SR (n = 8)	EoE (n = 16)	Normal Esophagus in EGD and Histopathology (n = 34)
I don’t know/I don’t remember [n (%)]	15 (25.86)	2 (28,57)	0	5 (31.25)	9 (26.47)
less than 1 week [n (%)]	7 (12.07)	0	1 (12.5)	2 (12.50)	5 (14.71)
from 1 week to 1 month [n (%)]	1 (1.72)	0	0	0	1 (2.94)
from 1 month to 6 months [n (%)]	5 (8.62)	0	0	2 (12.50)	3 (8.82)
over half a year [n (%)]	30 (51.72)	5 (71,43)	7 (87.50)	7 (43.75)	16 (47.06)

**Table 4:** Spearman’s rank correlation coefficients between diagnosis of ERD, SR, EoE, normal esophagus in EGD and histopathology examination and diagnostic delay of dysphagia. Statistically significant—in red.

Parameter	ERD	SR	EoE	Normal Esophagus in EGD and Histopathology
Diagnostic delay of dysphagia	0.093016	0.284778	−0.094162	−0.074057

**Table 5:** Spearman’s rank correlation coefficients between the diagnostic delay of dysphagia in ERD, SR, EoE, normal esophagus and manometric patterns and features. Significant—in red.

Esophageal Motility Disorders		Diagnostic Delay of Dysphagia in ERD	Diagnostic Delay of Dysphagia in SR	Diagnostic Delay of Dysphagia in EoE	Diagnostic Delay of Dysphagia in the Normal Esophagus in EGD and Histopathology
Motility pattern defined per Chicago classification	EGJ outflow obstruction	-	-	−0.327411	−0.177861
	Absent contractility	-	0.14286	0.267882	0.082995
	IEM	0.645497	−0.21822	−0.372058	−0.037459
	Normal peristalsis	−0.645497	0.14286	0.248705	0.097641
Manometric features	LES insufficiency	−0.300000	−1.00000	−0.714352	0.176476
	UES insufficiency	−0.300000	0.21822	−0.732114	0.183598
	UES spastic disorders	0.400000	−1.00000	−0.073837	0.036836
	Hiatal hernia	−0.300000	0.14286	−0.327411	−0.231161

**Table 6:** Spearman’s rank correlation coefficients between diagnosis of ERD, SR, EoE, normal esophagus and total GIQLI mean points in patients with or without esophageal motility disorders. Significant—in red.

	Esophageal Motility Disorders	ERD	SR	EoE	Normal Esophagus in EGD and Histopathology
**Total GIQLI Mean Points in Patients**	without EGJ outflow obstruction	0.169862	0.215819	0.352961	−0.182693
	with EGJ outflow obstruction	-	-	0.25820	−0.25820
	without absent contractility	0.151276	0.163310	0.325464	−0.144026
	with absent contractility	-	0.86603	0.86603	−0.86603
	without IEM	0.311293	0.162217	0.433543	−0.148147
	with IEM	0.086272	0.183328	0.277046	−0.165420
	without normal peristalsis	0.066890	0.200323	0.256506	−0.140999
	with normal peristalsis	0.346712	0.165819	0.519207	−0.192189
	without LES insufficiency	0.125747	0.271700	0.421602	−0.161055
	with LES insufficiency	0.295312	−0.247657	0.267964	−0.294093
	without UES insufficiency	−0.007154	0.068268	0.392817	−0.040431
	with UES insufficiency	0.675635	0.675635	−0.043483	−0.550019
	without UES spastic disorders	0.319678	0.204422	0.366977	−0.275286
	with UES spastic disorders	−0.202478	−0.218986	−0.085058	0.145546
	without hiatal hernia	0.155825	0.270708	0.303854	−0.207247
	with hiatal hernia	0.235702	−0.544331	0.816497	0.272166

**Table 7:** Spearman’s rank correlation coefficients between UES insufficiency and motility pattern defined per Chicago classification in patients with ERD, SR, EoE and normal esophagus in EGD and histopathology. Significant—in red.

Motility Pattern Defined per Chicago Classification	All Patients with UES Insufficiency	ERD with UES Insufficiency	SR with UES Insufficiency	EoE with UES Insufficiency	Normal Esophagus with UES Insufficiency
EGJ outflow obstruction	−0.063564	−0.188982	−0.188982	0.395285	−0.250000
Absent contractility	-	-	-	-	-
IEM	−0.225630	−0.357143	0.285714	−0.059761	−0.188982
Presence of any esophageal motility disorder defined per Chicago classification	−0.250000	0.357143	0.285714	−0.059761	−0.188982
Normal peristalsis	0.196221	0.357143	−0.285714	0.059761	0.188982

## Data Availability

The data generated and analyzed during the current study are available from the corresponding author upon reasonable request.

## Trial Registration

This trial was registered with ClinicalTrials.gov (no. NCT04803162). Registered 17 March 2021—retrospectively registered, https://clinicaltrials.gov/ct2/show/NCT04803162 (accessed on 6 September 2021).

## Abbreviations

BMI	body mass index;
DES	distal esophageal spasm;
EAT-10	Eating Assessment Tool-10;
EGD	esophagogastroduodenoscopy;
EGJ	esophagogastric junction;
EoE	eosinophilic esophagitis;
ERD	erosive reflux disease;
GIQLI	Gastrointestinal Quality of Life Index;
IEM	ineffective motility;
LES	lower esophageal sphincter;
NS	statistically non-significant;
SD	standard deviation;
SR	Schatzki ring;
UES	upper esophageal sphincter
